# Appraisal of Bioactive Compounds of Betel Fruit as Antimalarial Agents by Targeting Plasmepsin 1 and 2: A Computational Approach

**DOI:** 10.3390/ph14121285

**Published:** 2021-12-09

**Authors:** Fatimawali, Trina Ekawati Tallei, Billy Johnson Kepel, Mohammed Alorabi, Ahmed M. El-Shehawi, Widdhi Bodhi, Sefren Geiner Tumilaar, Ismail Celik, Gomaa Mostafa-Hedeab, Amany Abdel-Rahman Mohamed, Talha Bin Emran

**Affiliations:** 1 Pharmacy Study Program, Faculty of Mathematics and Natural Sciences, Sam Ratulangi University, Manado 95115, Indonesia; sefrentumilaar1@gmail.com; 2 The University Center of Excellence for Biotechnology and Conservation of Wallacea, Institute for Research and Community Services, Sam Ratulangi University, Manado 95115, Indonesia; 3 Department of Biology, Faculty of Mathematics and Natural Sciences, Sam Ratulangi University, Manado 95115, Indonesia; 4 Department of Chemistry, Faculty of Medicine, Sam Ratulangi University, Manado 95115, Indonesia; billy.kepel@unsrat.ac.id (B.J.K.); widdhibodhi@unsrat.ac.id (W.B.); 5 Department of Biotechnology, College of Science, Taif University, Taif 21944, Saudi Arabia; maorabi@tu.edu.sa (M.A.); elshehawi@hotmail.com (A.M.E.-S.); 6 Department of Pharmaceutical Chemistry, Faculty of Pharmacy, Erciyes University, Kayseri 38039, Turkey; ismailcelik@erciyes.edu.tr; 7 Pharmacology Department, Health Sciences Research Unit, Medical College, Jouf University, Sakaka 72446, Saudi Arabia; gomaa@ju.edu.sa; 8 Pharmacology Department, Faculty of Medicine, Beni-Suef University, Beni Suef 62521, Egypt; 9 Department of Forensic Medicine and Toxicology, Zagazig University, Zagazig 44519, Egypt; aabdaziz@zu.edu.eg; 10 Department of Pharmacy, BGC Trust University Bangladesh, Chittagong 4381, Bangladesh

**Keywords:** *Piper betle*, betel fruit, malaria, in silico, protease inhibitor, plasmepsin

## Abstract

In many countries, the fruit of betel (*Piper betle* Linn) is traditionally used as medicine for treating malaria. It is a fatal disease, and existing medications are rapidly losing potency, necessitating the development of innovative pharmaceutics. The current study attempted to determine the compounds in the n-hexane fraction of betel fruit extract and investigate the potential inhibition of bioactive compounds against aspartic protease plasmepsin 1 (PDB ID: 3QS1) and plasmepsin 2 (PDB ID: 1LEE) of *Plasmodium falciparum* using a computational approach. The ethanol extract was fractionated into n-hexane and further analyzed using gas chromatography-mass spectrometry (GC-MS) to obtain information regarding the compounds contained in betel fruit. Each compound’s potential antimalarial activity was evaluated using AutoDock Vina and compared to artemisinin, an antimalarial drug. Molecular dynamics simulations (MDSs) were performed to evaluate the stability of the interaction between the ligand and receptors. Results detected 20 probable compounds in the n-hexane extract of betel fruit based on GC-MS analysis. The docking study revealed that androstan-17-one,3-ethyl-3-hydroxy-, (5 alpha)- has the highest binding affinity for plasmepsin 1 and plasmepsin 2. The compound exhibits a similar interaction with artemisinin at the active site of the receptors. The compound does not violate Lipinski’s rules of five. It belongs to class 5 toxicity with an LD_50_ of 3000 mg/kg. MDS results showed stable interactions between the compound and the receptors. Our study concluded that androstan-17-one,3-ethyl-3-hydroxy-, (5 alpha)- from betel fruit has the potential to be further investigated as a potential inhibitor of the aspartic protease plasmepsin 1 and plasmepsin 2 of *Plasmodium falciparum*.

## 1. Introduction

Malaria is an infectious disease that is very widespread worldwide, affecting 100 countries with tropical and subtropical climates. Every year in the world, 300–500 million cases occur, resulting in 1–3 million deaths [1]. The disease is transmitted by mosquito vectors carrying unicellular parasites of the genus *Plasmodium*. Plasmodia are obligate intracellular parasites capable of infecting and replicating in erythrocytes following a silent replication phase in the liver. Four species (*P. falciparum*, *P. malariae*, *P. ovale*, and *P. vivax*) have traditionally been recognized as natural causes of human infection, but the recent increase in malaria cases caused by *Plasmodium knowlesi* in Southeast Asia has led physicians to consider it the fifth parasite that causes malaria in humans [2].

Today, the availability of safe, effective, practical, and economically affordable antimalarial drugs has improved, resulting in reduced mortality from the disease. Several drugs have been developed that inhibit or kill the asexual form of the parasite in human erythrocytes, such as quinine, chloroquine, pyrimethamine, sulfonamides, sulfones, and artemisinin derivatives. The problem is that these drugs have mostly failed in the healing process due to parasites that have become resistant to antimalarial drugs [3,4,5]. There are several reasons for the resistance of the parasites to these drugs. For example, parasites do not have an active site to bind chloroquine, so this drug cannot be concentrated in erythrocytes [6]. Various attempts have been made to develop more effective antimalarial drugs. One of them is through utilizing a computer simulation approach.

In *Plasmodium falciparum*, there are three different classes of proteases responsible for hemoglobin degradation, including aspartic proteases (plasmepsin I, II, IV, and HAP), cysteine proteases (falcipain-1, -2 and -3), and metalloproteases (falcilysin) [7]. Plasmepsin is synthesized in the form of an inactive precursor (membrane-bound proplasmepsin) and processed into mature plasmepsin and falcipain, which are categorized as cysteine proteases [8]. Because plasmepsin and falcipain are involved in the degradation of hemoglobin necessary for the proliferation of parasites in the body, they have been targets of anti-malarial drug development for decades [7,8]. Among these two proteins, plasmepsin is considered to be the ideal target for antimalarial drugs [9]. By targeting this protein, the parasite’s life cycle can be inhibited [10]. In addition, an antimalarial drug currently on the market, artemisinin, is targeted to inhibit the performance of both plasmepsin 1 and plasmepsin 2 [11].

The discovery of new drugs is labor-intensive and time-consuming. However, this process can at least be shortened through initial screening using a computational method. This strategy aims to improve the efficiency of the simulation and calculation procedures used in drug design, offering the in silico method as a complement to the in vitro and in vivo approaches that are frequently utilized in the process of drug discovery [12]. Using an in silico-based drug design approach, the difference in ligand and receptor bond energies is exploited between the target site of the parasite and the putative drug molecule. Stronger interactions displayed by some molecules compared to reference molecules represent potential drug candidates [7]. 

Several previous in silico studies have been conducted to identify antimalarial compounds. Bioactive flavonoid compounds from the roselle plant (*Hibiscus sabdariffa* L.) as an antimalarial compound against plasmepsin 1 and plasmepsin 2 produce a lower docking score than artemisinin, indicating that these compounds have better potential activity for the aspartic protease enzyme [13]. The results of pharmacological and molecular docking of phytol from *Moringa oleifera* demonstrated that this bioactive compound has potential as an antimalarial drug [14].

In pharmacological studies, the fruit and leaves of green betel (*Piper betel* Linn) have health and health-related benefits, including analgesia [15], anti-ulcer, anti-allergic [16], antibacterial [17,18,19,20], anti-mosquito larvae [21], antioxidant [22], and insect repellent properties [23]. Betel fruit has antimalarial activity in vivo in Wistar rats, which was administered by mixing it with mayana leaves, honey, and egg yolk [1]. This present study used the GC-MS method to determine the bioactive chemicals present in the n-hexane fraction of betel fruit grown in North Sulawesi. The identified compounds were then analyzed in silico to determine their potential for inhibiting the aspartic proteases plasmepsin 1 and 2 from *P. falciparum* and compared to artemisinin, a standard antimalarial drug.

## 2. Results

### 2.1. GC-MS Analysis

**Figure 1:**
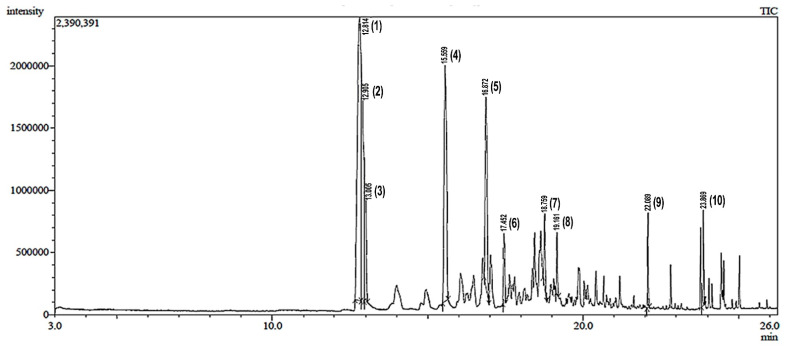
Total ionic chromatogram (TIC) of the n-hexane fraction of the betel fruit extract. The numbers (1)–(10) represent each peak, which corresponds to the information in Table 1.

According to GC-MS analysis, the n-hexane fraction of the betel fruit extract recorded a total of 10 readable peaks, corresponding to a total of 20 probable bioactive compounds (Figure 1). These compounds were recognized by relating their peaks’ retention times, peak area (%), height (%), and mass spectral fragmentation patterns to those of known compounds documented by the NIST (National Institute of Standards and Technology) mass spectra database and library. The chemical names of the bioactive compounds are shown in Table 1. Each retention time produces three probable compounds. There are some that produce the same probable compound, and some that are different, so all the probable compounds are considered results based on the database used.

### 2.2. ADME Analysis

All probable compounds detected were used as ligands and then examined for their pharmacokinetic properties using ADME (absorption, distribution, metabolism, and excretion) tools. Table 2 summarizes the findings. According to this finding, all the probable compounds detected in betel fruit comply with Lipinski’s rule of five (Ro5), except neoclovenoxid-alcohol, which indicates that these compounds would be likely orally active medicine in humans. The Ro5 criteria are as follows: (i) the molecular weight is ≤500 g/mol; (ii) the number of H-bond acceptors is ≤10; (iii) the number of H-bond donors is ≤5; (iv) the log *p*-value (lipophilicity) is ≤5; and (v) the molar refractivity should be between 40 and 130.

### 2.3. Toxicity Analysis

Several parameters in the toxicity analysis included LD_50_, predicted toxicity class, hepatoxicity, carcinogenicity, immunotoxicity, mutagenicity, and cytotoxicity. Toxicity levels are classified as follows: classes 1 and 2 (fatal if swallowed), class 3 (toxic if swallowed), class 4 (harmful if swallowed), class 5 (maybe harmful if swallowed), and class 6 (non-toxic). As shown in Table 3, based on their respective LD_50_ (mg/kg), seven compounds are classified as harmful if swallowed (class 4), one compound is non-toxic (class 6), and the remaining compounds may be harmful if swallowed (class 5). None of the compounds demonstrated mutagenicity or cytotoxicity. Benzoic acid, 2,4-dimethyl- and benzoic acid, 2,6-dimethyl- are predicted to have hepatotoxicity activity with a probability of 0.52. Guaicoal is predicted as carcinogenic with a probability of 0.56. Androstan-17-one, ethyl-3-hydroxy-, (5-alpha)-, Torreyol, longipinocarveol, trans-, alpha-Cadinol, and artemisinin all demonstrated significant immunotoxicity, with probabilities of 0.79, 0.69, 0.62, 0.69, and 0.70, respectively.

### 2.4. Molecular Docking Analysis

The docking results revealed that some of the bioactive compounds from the fractionation of n-hexane betel fruit exhibited substantial binding-free energy (BFE) values compared to artemisinin (Table 4). The BFE value of the compounds ranged from −4.7 to −9.1 kcal/mol for plasmepsin 1, and from −4.5 to −8.0 kcal/mol for plasmepsin 2. Meanwhile, artemisinin, which acted as a control, exhibited a BFE value of −7.7 and −6.7 kcal/mol for plasmepsin 1 and 2, respectively.

**Figure 2:**
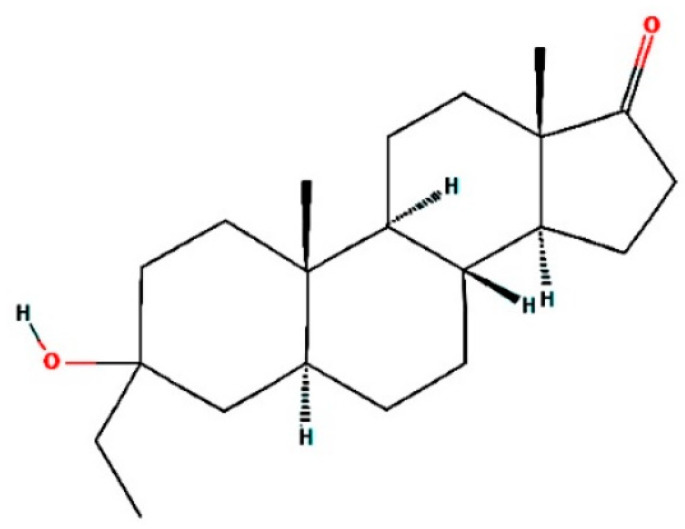
The two-dimensional structure of androstan-17-one, ethyl-3-hydroxy-, (5-alpha)-.

**Figure 3:**
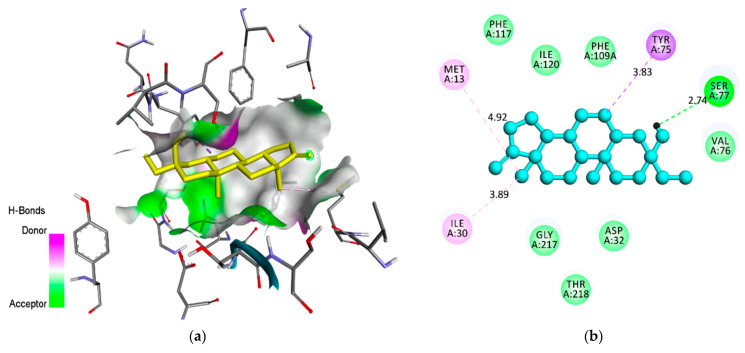
A molecular interaction between AND and 3QS1: (**a**) AND’s binding position in the active site of 3QS1; (**b**) the type of interaction of AND that binds to the amino acids of 3QS1.

**Figure 4:**
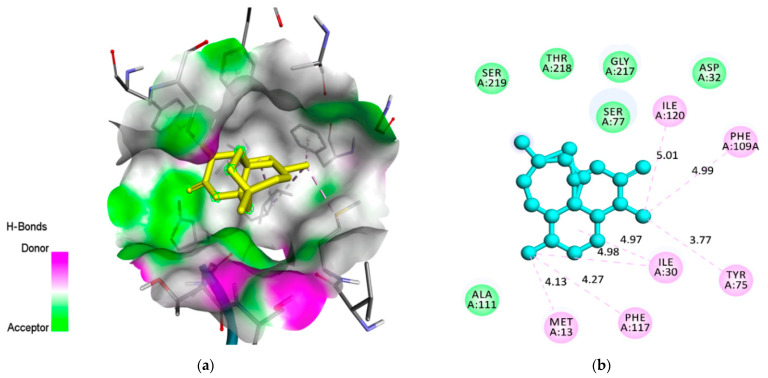
A molecular interaction between artemisinin and 3QS1: (**a**) artemisinin’s binding position in the active site of 3QS1; (**b**) the type of interaction of artemisinin that binds to the amino acids of 3QS1.

The compound androstan-17-one, ethyl-3-hydroxy-, (5 alpha)- (hereafter referred to as AND) (Figure 2), which showed the best results for 3QS1 and 1LEE, was selected for molecular interaction analysis (Table 5 and Table 6). For comparison, the molecular interactions between artemisinin, acting as a control, and 3QS1 and 1LEE are shown in Table 5. Results of the investigation of molecular interactions revealed that AND interacts with residues MetA13, IleA30, SspA32, TyrA75, ValA75, SerA77, PheA109, PheA117, IleA120, GlyA2178, and ThrA21 with 3QS1 (Figure 3). Meanwhile, the interactions shown by artemisinin with the same receptor are with residues MetA13, IleA30, AspA32, TyrA75, SerA77, PheA109, AlaA111, PheA117, IleA120, GlyA217, ThrA218, and SerA219 (Figure 4). It appears that these two compounds bind to the same site of 3QS1, albeit through different types of interactions and at different distances.

**Figure 5:**
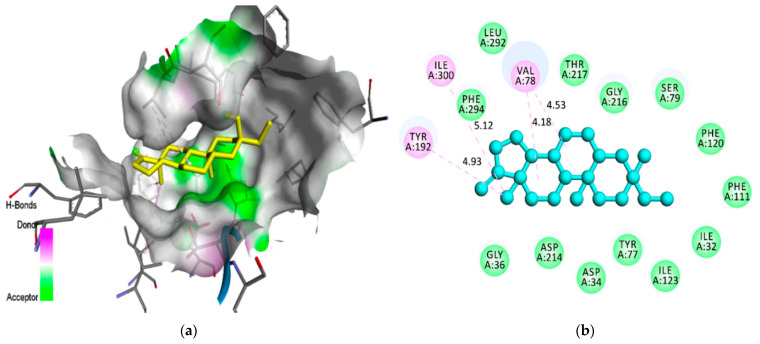
A molecular interaction between AND and 1LEE: (**a**) AND’s binding position in the active site of 1LEE; (**b**) the type of interaction of AND that binds to the amino acids of 1LEE.

**Figure 6:**
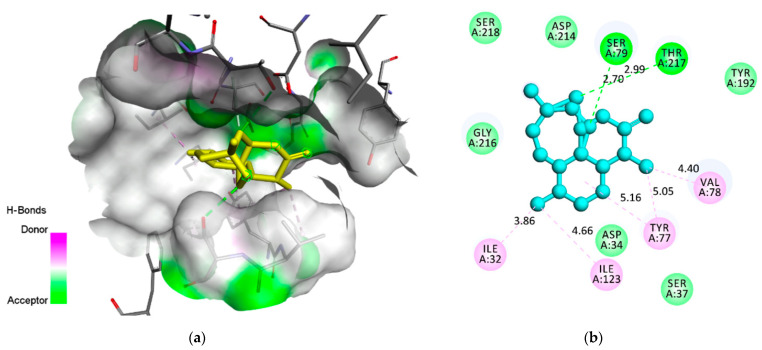
**A** molecular interaction between artemisinin and 1LEE: (**a**) artemisinin’s binding position in the active site of 1LEE; (**b**) the type of interaction of artemisinin that binds to the amino acids of 1LEE.

With the 1LEE receptor, AND interacts at residues IleA32, AspA34, GlyA36, TyrA77, ValA78, SerA79, PheA111, PheA120, TyrA192, IleA123, AspA214, GlyA216, ThrA217, LeuA292, PheA294, and IleA300 (Figure 5). Meanwhile, the control ligand artemisinin binds to the ILEE receptor at residues IleA32, AspA34, SerA37, SerA79, TyrA77, ValA78, IleA123, TyrA192, AspA214, GlyA216, ThrA217, and SerA218 (Figure 6). These two compounds appear to share a common binding site, although there are some differences in the binding of the residues and the types of interactions. The binding of AND to the same pocket residue as artemisinin, both to plasmepsin 1 and 2, indicates that AND has potential as an inhibitor for these two receptors.

### 2.5. Molecular Dynamics Simulation

Interactions between AND and 3QS1 were simulated for 50 ns. First, the interaction between AND and 3QS1 was analyzed concerning the protein–ligand complex. Based on the protein backbone atoms, the root-mean-square deviation (RMSD) was calculated to measure changes in the 3QS1 apo form (3QS1-Apo), 3QS1-AND complex (3QS1-AND), and AND (AND-AND) over the time of the simulation. As shown in Figure 6, the 3QS1-AND complex deviated very little and remained constant throughout the simulation. The average RMSD values of 3QS1-Apo, 3QS1-AND, and AND-AND were measured at 0.229 nm, 0.279 nm, and 0.053 nm, respectively.

**Figure 7:**
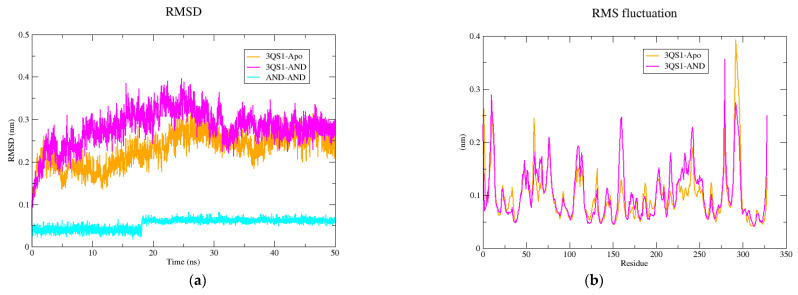

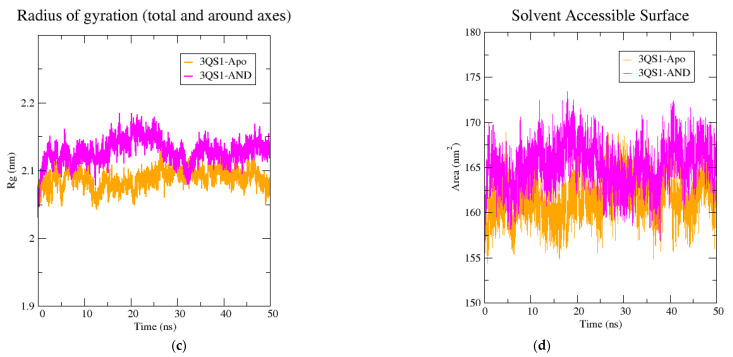
Molecular dynamics simulations analysis: (**a**) root-mean-square deviation (RMSD); (**b**) root-mean-square fluctuation (RMSF); (**c**) radius of gyration (Rg); and (**d**) solvent accessible surface area (SASA) graphs of the apo form (3QS1-Apo) and holo form (3QS1-AND) over 50 ns.

According to the RMSF analysis shown in Figure 7, the binding of AND to 3QS1 did not negatively alter the fluctuation or stability of the protein. Between the 285th and 289th amino acids, where the apo form showed the highest fluctuation, 3QS1-Apo peaked at 0.39 nm, while the 3QS1-AND holo form peaked at 0.27 nm.

Another method used in the evaluation of protein compactness is the measurement of radius of gyration (Rg) values. The smaller the Rg values and the fewer deviations they explain, the higher the compactness of the protein. As shown in Figure 7, 3QS1-Apo and 3QS1-AND Rg values with small fluctuations between 2.05 nm and 2.17 nm were obtained. The solvent accessible surface area (SASA) measurements are used to understand changes in the protein’s solvent accessible surface area and stability as a result of the interaction of the ligand with the protein. For this purpose, SASA analysis of the apo form of 3QS1 and the AND-linked holo form was performed. As shown in Figure 7, average SASA values for 3QS1-Apo and 3QS1-AND were 161.49 nm^2^ and 165.19 nm^2^ SASA, respectively. The binding of AND to 3QS1 increased the average SASA value of the protein.

**Figure 8:**
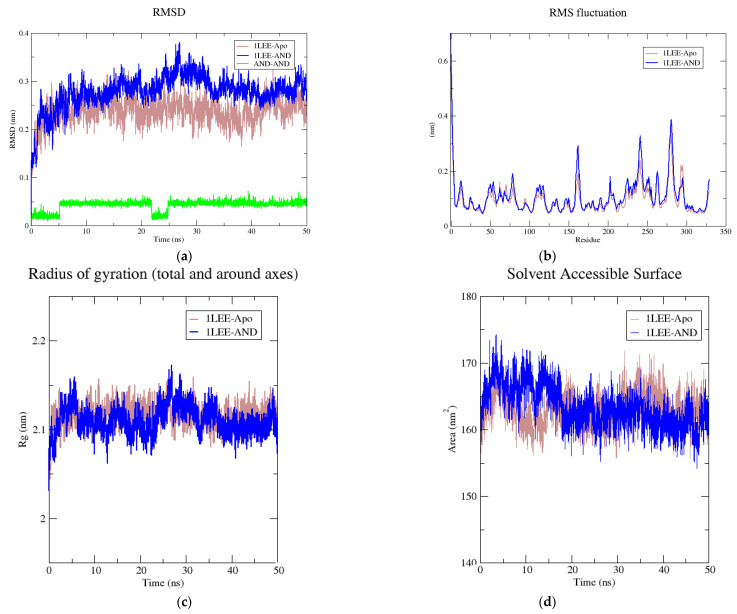
Molecular dynamics simulation of AND with antimalarial target plasmepsin 2; (**a**) RMSD of apo-(1LEE-Apo) and ligand-bound plasmepsin 2 (1LEE-AND), (**b**) RMS fluctuation, (**c**) Rg, and (**d**) SASA values during the period of 50 ns simulation.

**Figure 9:**
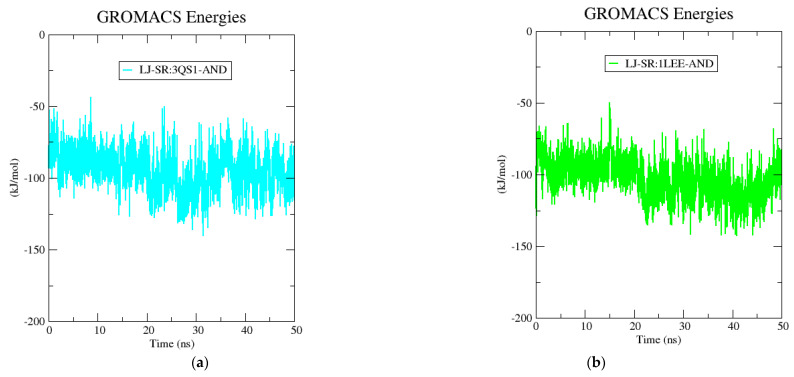
Short-range Lennard-Jones protein–ligand interaction energy between (**a**) 3QS1-AND, and (**b**) 1LEE-AND, and AND compounds for 50 ns.

The second trajectory analysis was performed to analyze the interaction between AND and 1LEE and their changes over the simulation time. An RMSD analysis was performed to examine changes in protein stability over time. As illustrated in Figure 8, the root-mean-square deviation (RMSD) values for the 1LEE-AND complex was determined to be less than 0.4 nm. The average RMSD values of 1LEE-Apo, 1LEE-AND, and AND-AND were measured at 0.236 nm, 0.277 nm, and 0.042 nm, respectively. According to the root-mean square fluctuation (RMSF) analysis performed to measure protein fluctuation, 1LEE-Apo and 1LEE-AND exhibited quite similar conformational changes, as shown in Figure 8. The interaction between AND and 1LEE did not impair protein stability. According to the radius of gyration (Rg) analysis performed to measure the compactness of 1LEE-Apo and 1LEE-AND, Rg values between 2.05 nm and 2.17 nm were observed. Based on solvent accessible surface area (SASA) analysis to evaluate the solvent-accessible area of the protein–ligand complex, 1LEE-Apo and 1LEE-AND yielded average values of 163.13 and 163.42 nm^2^, respectively. In this study, time-dependent changes in the short-range Lennard-Jones energy between AND and 3QS1-1LEE and the energy of the environment over 50 ns were calculated. As shown in Figure 9, the stable binding energy of AND was measured in both 3QS1-AND and 1LEE-AND. The 3QS1-AND and 1LEE-AND protein–ligand complexes formed average short-range Lennard-Jones energies of −95.2164 and −102.94 kJ mol^−1^, respectively.

## 3. Discussion

Malaria is a public health problem in developing countries that can cause death, especially in high-risk groups. Due to drug resistance in the treatment of the disease with numerous medications, it is vital to look for promising medicinal plants in traditional anti-malarial medicine that have been scientifically tested. Traditional Indonesian medicinal plants are a potential source of novel antimalarial substances. One of them is the fruit of the betel plant (*P. betle*). In this study, we used the fractionation of n-hexane from a methanol extract of betel fruit in an effort to identify anti-malarial drugs. GC-MS analysis yielded 20 probable compounds, which are shown in Figure 1 and detailed in Table 1. Generally, these compounds are reported as antimicrobials. Table 1 shows that the compounds with the highest number in the n-hexane fraction of betel fruit are located at peak 1 with a percent content (retention area) of 32.22%. The three compounds present at peak 1 are the same: phenol, 2-methoxy-3- (2-propenyl)-. The second-largest component is observed at peak 4, with a percentage of 18.86%. The three compounds at peak four are also the same compound: benzoic acid, 2,4-dimethyl-. All of the probable compounds detected by GC-MS were used as ligands in molecular docking.

The aim of searching for ligand-based drugs is to identify ligands that can interact effectively with the target receptors. However, this does not mean that the compound will be active if given orally. The journey to drug targets and drug interactions in the body consists of pharmacokinetic events, including ADME. Therefore, it is necessary to consider pharmacokinetics in the design of new drugs [24]. When designing an orally active drug, it should meet the criteria of Lipinski’s Ro5 [25,26]. This rule is used to establish whether particular chemical compounds possess the requisite chemical and physical qualities for usage as active pharmaceutical ingredients that may be administered orally to humans and to evaluate drug similarities [25]. Lipinski’s Ro5 analysis demonstrates that the five probable compounds with the highest BFE have excellent bioavailability due to their compliance with the rules. As a result, these compounds are predicted to be active when administered orally. This means that the compound easily binds to the receptor and that the ligand can cross the cell membrane easily [27].

In modern drug discovery, one of the most important components is the toxicity prediction of potential drug candidates. This includes hepatotoxicity, carcinogenicity, immunotoxicity, mutagenicity, and cytotoxicity, which are the most important factors to consider when searching for new drugs with potentially beneficial properties. Acute toxicity of a compound is expressed as a median lethal dose (LD_50_) [28]. The LD_50_ of the compounds studied ranged from 2000 (class 4 toxicity) to 5000 (class 5 toxicity) mg/kg. In general, the lower the LD_50_, the more toxic the substance [29]. Only a few compounds under study, including artemisinin, have the potential to be immunotoxic. Immunotoxicity is described as the immune system’s maladaptive functioning after exposure to a xenobiotic chemical. These events include immune system dysfunction and hyperactivity, resulting in cellular damage and permanent or reversible changes in the immune response [30].

The compounds that passed the pharmacokinetic and toxicity screening were further analyzed for their binding affinity for plasmepsins. The enzymes are aspartic proteases that work in coordination with cysteine proteases to degrade hemoglobin in the parasite’s food vacuole [31]. These enzymes have been identified as possible targets for the development of new antimalarial drugs [9]. This study suggests that AND has a favorable interaction with plasmepsin 1 and 2, based on their BFE values being greater than those of artemisinin. Artemisinin, as a positive control in this study, inhibits plasmepsins and represents an antimalarial agent [11].

The receptors 3QS1 and 1LEE have the native ligands KNI and R36, respectively. The native ligands were extracted and redocked into their original binding pockets. The RMSD analysis is one of the most widely used parameters for calculating protein atomic deviations [32]. The RMSD values resulting from these superimposing native ligands after redocking to their original binding pockets were 1.7099 Å and 1.2918 Å. These values are <2.0000 Å, a value typically used in evaluating the success of docking algorithms. This indicates that the docking method was valid [13]. The binding positions of AND and artemisinin on plasmepsins were in the same pocket, suggesting that AND provides new hope as an antimalarial lead candidate. As a result, further visualization and molecular dynamics simulation were performed.

The three parameters that are usually considered when calculating molecular docking results are binding affinity, the interaction of the amino acid residuals involved, and the hydrogen bond energy [33]. Several amino acid residues are involved in the binding of the compounds to plasmepsins, and the presence of hydrogen bonds is involved so that these three parameters stabilize AND bonds at plasmepsins, indicating that this compound inhibits the activity of these enzymes. The interactions that occur between artemisinin as a ligand and amino acid residues in the receptors demonstrate that artemisinin as a positive control ligand interacts with 10 amino acid residues of the receptor 3QS1 and 14 amino acid residues of the receptor 1LEE. According to the type of AND interaction with 3QS1 and 1LEE, the interactions involve 11 amino acid residues in 3QS1 and 15 amino acid residues in 1LEE, implying that the interactions involve more amino acids than artemisinin. The accuracy of ligand binding to the receptor can be seen from the amino acid residues that interact with ligand and receptor binding [34].

Molecular dynamics simulation studies in drug active ingredient designs are frequently used in predictive studies of potential ligand–receptor interactions [35]. In an in silico physiological environment, simulations are accepted as a rational approach for evaluating the molecular dynamics and interactions between the ligand and the protein [36]. From this point of view, the change in AND over time was investigated and analyzed by the molecular docking of plasmepsins. A molecular dynamics simulation of 50 ns duration was performed using CHARMM force fields, and RMSD, RMSF, Rg, and SASA trajectory analysis were measured for both target proteins.

Measuring the binding energy between protein and ligand against time is one of the important approaches in molecular dynamics simulations where protein–ligand interactions are investigated [37]. Lennard-Jones energy measurements are one of the most widely used molecular dynamics simulations to measure the potential energy of two molecules that interact but do not bond with each other [38]. Hence, the short-range Lennard-Jones protein–ligand interaction energy was calculated. To investigate the effect of AND on two target proteins, the ligand-free apo form was simulated in the same environment and conditions. When the data obtained from molecular dynamics simulations, RMSD, RMSF, Rg, SASA, and the average short-range Lennard-Jones energy were evaluated, the stability of the complex in which AND forms plasmepsin 1 and plasmepsin 2 proteins was evaluated. The ligand gave very small deviations in the complex of AND formed by plasmepsin 1 and plasmepsin 2. It is also understood that the protein–ligand Lennard-Jones energy is a constant interaction throughout the simulation.

The results of a pharmacoinformatics study of potential compounds of betel fruit indicate that these compounds have significant implications in the search for antimalarial drugs. As public trust in the applicability and reliability of in silico approaches grows, so will their use in regulatory decision-making [39].

## 4. Materials and Methods

### 4.1. Plant Collection

Ripe betel fruits were collected from Kotamobagu City, North Sulawesi, Indonesia. The fruits were washed under running water and dried in a drying cabinet at 45 °C for 24 h. The dried fruits were ground into powder using a mixer grinder, passed through a 40-mesh Sieve to achieve a very fine powder, and stored in an airtight container until used.

### 4.2. Sample Preparation

Ten grams of dried betel fruit powder were macerated in 100 mL of 95% ethanol for three days with occasional shaking and filtered using Whatman No. 1 filter paper. Then, the supernatant was evaporated using a rotary vacuum evaporator to obtain a concentrated extract. The extract was subsequently fractionated into ethyl acetate and n-hexane. The obtained n-hexane solution was centrifuged, and the supernatant was used in further analysis.

### 4.3. Gas Chromatography-Mass Spectrometer (GC-MS) Analysis

Compound analysis was performed using GC-MS (Shimadzu QP 2010 SE). This GC-MS uses an electron ionizing system (EI) with helium as the carrier gas at a constant flow rate of 1 mL/minute for a total time of 60 min at a temperature of 280 °C. The column was a DB-1 (100% dimethylpolysiloxane) with a length of 30 m and a diameter of 0.25 mm. The temperature of the column was set at 40–270 °C, with a temperature increase of 10 °C every 5 min.

### 4.4. In Silico ADMET Analysis

The studied compounds underwent pharmacokinetic and drug-like as well as toxicity analysis. The pharmacokinetic properties and drug-like nature were predicted by the Supercomputing Facility for Bioinformatics and Computational Biology (SCFBIO) (http://www.scfbio-iitd.res.in/software/drugdesign/lipinski.jsp; accessed on 23 July 2021) [40]. The toxicity prediction was carried out on ProTox-II (https://tox-new.charite.de/protox_II/; accessed on 23 July 2021) [41].

### 4.5. Computational Molecular Docking Analysis

#### 4.5.1. Preparation of the Receptors

The receptor preparation procedure followed the steps of Tallei et al. [42]. The plasmepsin-1 (PDB ID: 3QS1) and plasmepsin-2 (PDB ID: 1LEE) macromolecules, which serve as receptors for targets in molecular docking, were downloaded from the Protein Databank (http://www.rscb.org/pdb/; accessed on 29 July 2021). These receptors were opened using BIOVIA Discovery Studio Visualizer 2020 and separated from solvents and nonstandard ligands or residues. The cleaned receptors were stored in pdb format and used for the docking process, and then optimized using Autodock Tools [43]. Optimization included adding hydrogen atoms and setting the grid box parameter. These results were saved in pdbqt.

#### 4.5.2. Preparation of the Ligands

The ligands used were bioactive compounds isolated from betel leaves that were produced by the GC-MS analysis and the artemisinin drug as a control. The structures of these ligands were downloaded from http://pubchem.ncbi.nlm.nih.gov (accessed on 29 July 2021) in the sdf format. The procedure for ligand preparation followed the steps by Tumilaar [44]. The file format of these ligands was converted to pdb using Open Babel [45] and optimized using Autodock Tools [43]. The optimization includes setting the number of active torsions. The optimization results were saved in pdbqt format.

#### 4.5.3. Molecular Docking

Molecular docking steps followed the procedure of Sailah et al. [46]. Ligands and receptors in the pdbqt format were copied into the vina folder. The Vina configuration file was typed in notepad and saved as ‘conf.txt’. Vina was run via the command prompt. The docking calculation results were viewed in the output in notepad format. Determination of the ligand conformation resulting from docking was performed by selecting the ligand conformation that had the lowest binding free energy (best pose). The binding free energy value was displayed in the log.txt file. The position and orientation of the ligands on the receptor macromolecules and the amino acids bound to the ligands were visualized using BIOVIA Discovery Studio Visualizer 2020 software. Receptor and ligand files in pdbqt format were displayed in the application, and then the ligand–receptor interactions were displayed in 2D and 3D conformations.

### 4.6. Molecular Dynamics Simulation

Molecular dynamics simulations to investigate protein–ligand stability and interaction energy were performed using the GROningen MAchine for Chemical Simulations (GROMACS) [47] according to the protocol of Celik et al. [48]. The androstan-17-one, 3-ethyl-3-hydroxy-, (5 alpha)–ligand topology file was created with the CGenFF server (https://cgenff.umaryland.edu/; accessed on 14 August 2021) and the topology file of plasmepsin 1 and 2 protein structures with the pdb2 gmx script using the Charmm36-Jul2020 force field [49]. System energy was minimized, and canonical ensembles (amount of substance (N), pressure (P), and temperature (T)-NVT) and isothermal-isobaric ensembles (amount of substance (N), volume (V), and equilibrium step temperature (T)-NPT) were performed at 0.1 ns and 1 ns, respectively. Molecular dynamics simulations of a standard 50 ns duration were performed. The RMSD, RMSF Rg, and SASA analyses were performed. Finally, the average interaction energy between the protein and ligand was calculated according to the short-range Lennard-Jones energy. All trajectory analysis graphics were created using QtGrace tools.

## 5. Conclusions

This in silico study evaluated the inhibition activity of betel fruit compounds against plasmepsins, which are aspartic proteases found in the malaria parasite *P. falciparum*. The compound androstan-17-one, ethyl-3-hydroxy-, (5 alpha) (referred to as AND) exhibited the highest binding affinity against plasmepsins. Additionally, the compound exhibited hydrogen bond, hydrophobic, and electrostatic interactions with the receptors, indicating that these compounds interact strongly with plasmepsins. Furthermore, the AND binding site on the receptor was right at the active site of plasmepsins, indicating that this compound could inhibit plasmepsin activity. Dynamics simulations study validated the stability of the bond between AND and plasmepsins. However, the present work is only an initial screening to facilitate further research on the potential of AND as a lead compound. Furthermore, beyond a pharmacoinformatics approach, in vivo proof-of-concept testing is required to ensure that the proposed compound is truly effective against malarial proteases while not targeting human proteases.

## Figures and Tables

**Table 1:** Compounds from the n-hexane fraction of betel fruit extract identified using GC-MS analysis.

Peak	Retention Time (min)	Probable Compound Name #Hit1	Probable Compound Name #Hit2	Probable Compound Name #Hit3	Retention Area (%)
1	12.814	Phenol, 2-methoxy-3-(2-propenyl)-	Phenol, 2-methoxy-4-(2-propenyl)-	Phenol, 2-methoxy-4-(2-propenyl)-	32.22
2	12.905	4-Nitroisopropylbenzene	4-Nitroisopropylbenzene	3-Nitroisopropylbenzene	16.99
3	13.005	Guaiacol, 3-allyl-	p-Eugenol	p-Eugenol	7.10
4	15.559	Benzoic acid, 2,4-dimethyl-	Benzoic acid, 2,4-dimethyl-	Benzoic acid, 2,6-dimethyl-	18.86
5	16.872	Delta-Cadinene	delta-Cadinene	delta-Cadinene	11.85
6	17.452	Nerolidol	Nerolidol b (cis or trans)	d-Nerolidol	3.04
7	18.759	alpha-Cadinol	Epiglobulol	Torreyol	2.84
8	19.161	Androstan-17-one, 3-ethyl-3-hydroxy-, (5 alpha)-	Longipinocarveol, trans-	Neoclovenoxid-alcohol	1.95
9	22.089	Hexadecanoic acid, methyl ester	Hexadecanoic acid, methyl ester	Hexadecanoic acid, methyl ester	2.37
10	23.869	9-Octadecenoic acid, methyl ester	9-Octadecenoic acid (Z)-, methyl ester	9-Octadecenoic acid (Z)-, methyl ester	2.78

**Table 2:** Lipinski’s rule of the plasmepsin protease potential inhibitors.

Ligand Properties	PubChem ID	Mol. Weight < 500 g/mol	No. H-Bond Donors < 5	No. H-Bond Acceptors < 10	Log *p* < 5	No. of Violation
Androstan-17-one, ethyl-3-hydroxy-, (5 alpha)-	14681481	318.50	1	2	4.4	0
Torreyol	11990360	222.37	1	1	3.3	0
Delta-cadinene	12306054	204.35	0	0	3.8	0
Epiglobulol	11858788	222.37	1	1	3.7	0
Longipinocarveol, trans-	534645	220.35	1	1	3.8	0
Alpha-Cadinol	6431302	223.37	3	5	3.78	0
Neoclovenoxid-alcohol	16211877	220.35	1	6	3.22	1
9-Octadecenoic acid, methyl ester	5280590	34.06	1	1	0.57	0
d-Nerolidol	5356544	194.31	1	1	3.54	0
Nerolidol	5284507	222.37	1	1	4.19	0
Benzoic acid, 2,4-dimethyl-	11897	150	1	2	2	0
Nerolidol b (cis or trans)	131753171	233.26	1	3	4.5	0
Eugenol	3314	164	1	2	2.2	0
3-Nitroisopropylbenzene	591251	165.19	0	2	2.07	0
4-Nitroisopropylbenzene	15749	165	0	2	2.12	0
Benzoic acid, 2,6-dimethyl-	12439	150	1	2	2.3	0
Phenol, 2-methoxy-3-(2-propenyl)-	596373	125	1	2	2.98	0
Phenol, 2-methoxy-4-(2-propenyl)-	3313	125	1	3	2.9	0
Hexadecanoic acid, methyl ester	8181	270	0	2	5.6	0
Guaicoal	460	312	5	6	0.05	0
Artemisinin (control)	68827	282.33	0	5	2.8	0

**Table 3:** Toxicity analysis of the five plasmepsin protease potential inhibitors.

Compounds	LD_50_ (mg/kg)	Predicted Toxicity Class	Hepatotoxicity (Prediction/ Probability)	Carcinogenicity (Prediction/ Probability)	Immuno- Toxicity (Prediction/ Probability)	Mutagenicity (Prediction/ Probability)	Cytotoxicity (Prediction/ Probability)
Androstan-17-one, ethyl-3-hydroxy-, (5-alpha)-	3000	5	−/0.52	−/0.78	+/0.79	−/0.96	−/0.82
Torreyol	2830	5	−/0.82	−/0.66	+/0.69	−/0.91	−/0.87
Delta-cadinene	4390	5	−/0.82	−/0.75	−/0.68	−/0.68	−/0.69
Epiglobulol	2000	4	−/0.77	−/0.69	−/0.87	−/0.75	−/0.89
Longipinocarveol, trans-	5000	5	−/0.89	−/0.64	+/0.62	−/0.92	−/0.96
Alpha-Cadinol	2830	5	−/0.82	−/0.66	+/0.69	−/0.91	−/0.87
Neoclovenoxid-alcohol	2000	4	−/0.77	−/0.75	−/0.94	−/0.75	−/0.86
9-Octadecenoic acid, methyl ester	3000	5	−/0.59	−/0.56	−/0.96	−/0.98	−/0.70
d-Nerolidol	5000	5	−/0.81	−/0.65	−/0.99	−/0.91	−/0.81
Nerolidol	5000	5	−/0.81	−/0.65	−/0.99	−/0.91	−/0.81
Benzoic acid, 2,4-dimethyl-	3200	5	+/0.52	−/0.72	−/0.99	−/0.97	−/0.88
Nerolidol b (cis or trans)	5000	6	−/0.75	−/0.66	−/0.99	−/0.92	−/0.79
Eugenol	1930	4	−/0.67	−/0.73	−/0.83	−/0.97	−/0.90
3-Nitroisopropylbenzene	430	4	−/0.51	−/0.52	−/0.86	−/0.57	−/0.79
4-Nitroisopropylbenzene	1000	4	−/0.51	−/0.52	−/0.96	−/0.57	−/0.79
Benzoic acid, 2,6-dimethyl-	4480	5	+/0.52	−/0.72	−/0.99	−/0.97	−/0.88
Phenol, 2-methoxy-3-(2-propenyl)-	1230	4	−/0.68	−/0.72	−/0.70	−/0.84	−/0.86
Phenol, 2-methoxy-4-(2-propenyl)-	916	4	−/0.74	−/0.62	−/0.70	−/0.84	−/0.86
Hexadecanoic acid, methyl ester	5000	5	−/0.58	−/0.55	−/0.90	−/0.83	−/0.70
Guaicoal	520	4	−/0.72	+/0.56	−/0.85	−/0.99	−/0.81
Artemisinin	4228	5	−/0.72	−/0.63	+/0.70	−/0.63	−/0.97

**Table 4:** Binding free energy of bioactive compounds of the betel fruit extract.

Ligand Properties	Binding Free Energy (kcal/mol)	
	1LEE (Plasmepsin 2)	3QS1 (Plasmepsin 1)
Androstan-17-one, ethyl-3-hydroxy-, (5-alpha)-	−8.0	−9.1
Torreyol	−6.6	−6.4
Delta-cadinene	−6.4	−6.3
Epiglobulol	−6.4	−6.3
Longipinocarveol, trans-	−6.1	−7.1
Alpha-Cadinol	−6.0	−6.1
Neoclovenoxid-alcohol	−6.0	−6.0
9-Octadecenoic acid, methyl ester	−5.9	−5.8
d- Nerolidol	−5.8	−6.1
Nerolidol	−5.8	−6.1
Benzoic acid, 2,4-dimethyl-	−5.6	−5.6
Nerolidol b (cis or trans)	−5.4	−5.6
Eugenol	−5.4	−5.5
3-Nitroisopropylbenzene	−5.3	−6.0
4-Nitroisopropylbenzene	−5.2	−5.8
Benzoic acid, 2,6-dimethyl-	−5.0	−5.1
Phenol, 2-methoxy-3-(2-propenyl)-	−5.0	−5.3
Phenol, 2-methoxy-4-(2-propenyl)-	−4,9	−5.0
Hexadecanoic acid, methyl ester	−4.9	−4.9
Guaicoal	−4.5	−4.7
Artemisinin (control)	−6.7	−7.7

**Table 5:** Molecular interactions of AND with 3QS1 and 1LEE.

Receptor Name	Binding Affinity (kcal/mol)	No. H-Bond	Interacting Residues	Distance (Å)	Category	Type of Interaction
Plasmepsin 1 (3QS1)	−9.1	1	Ser(A77)	2.74	H-Bond	Conventional
			Tyr(A75)	3.83	Hydrophobic	Pi-Sigma
			Met(A13)	4.92	Hydrophobic	Alkyl
			Ile(A30)	3.89	Hydrophobic	Alkyl
			Phe(A117)	-	Electrostatic	Van der Waals
			Ile(A120)	-	Electrostatic	Van der Waals
			Phe(A109)	-	Electrostatic	Van der Waals
			Val(A76)	-	Electrostatic	Van der Waals
			Asp(A32)	-	Electrostatic	Van der Waals
			Thr(A218)	-	Electrostatic	Van der Waals
			Gly(A217)	-	Electrostatic	Van der Waals
Plasmepsin 2 (1LEE)	−8	0	Ile(A300)	5.12	Hydrophobic	Pi-Alkyl/Alkyl
			Val(A78)	4.18	Hydrophobic	Pi-Alkyl/Alkyl
			Val(A78)	4.53	Hydrophobic	Pi-Alkyl/Alkyl
			Tyr(A192)	4.93	Hydrophobic	Pi-Alkyl/Alkyl
			Gly(A36)	-	Electrostatic	Van der Waals
			Asp(A214)	-	Electrostatic	Van der Waals
			Asp(A34)	-	Electrostatic	Van der Waals
			Tyr(A77)	-	Electrostatic	Van der Waals
			Ile(A123)	-	Electrostatic	Van der Waals
			Ile(A32)	-	Electrostatic	Van der Waals
			Phe(A111)	-	Electrostatic	Van der Waals
			Phe(A120)	-	Electrostatic	Van der Waals
			Ser(A79)	-	Electrostatic	Van der Waals
			Gly(A216)	-	Electrostatic	Van der Waals
			Thr(A217)	-	Electrostatic	Van der Waals
			Leu(A292)	-	Electrostatic	Van der Waals
			Phe(A294)	-	Electrostatic	Van der Waals

**Table 6:** Molecular interactions of artemisinin with 3QS1 and 1LEE.

Receptor Name	Binding Affinity (kcal/mol)	No. H-Bond	Interacting Residues	Distance (Å)	Category	Type of Interaction
Plasmepsin 1 (3QS1)	−7.7	0	Ile(A120)	5.01	Hydrophobic	Pi-Alkyl/Alkyl
			Phe(A109)	4.99	Hydrophobic	Pi-Alkyl/Alkyl
			Tyr(A75)	3.77	Hydrophobic	Pi-Alkyl/Alkyl
			Ile(A30)	4.97	Hydrophobic	Pi-Alkyl/Alkyl
			Ile(A30)	4.98	Hydrophobic	Pi-Alkyl/Alkyl
			Phe(A117)	4.27	Hydrophobic	Pi-Alkyl/Alkyl
			Met(A13)	4.13	Hydrophobic	Pi-Alkyl/Alkyl
			Ala(A111)	-	Electrostatic	Van der Waals
			Ser(A219)	-	Electrostatic	Van der Waals
			Thr(A218)	-	Electrostatic	Van der Waals
			Gly(A217)	-	Electrostatic	Van der Waals
			Ser(A77)	-	Electrostatic	Van der Waals
			Asp(A32)	-	Electrostatic	Van der Waals
Plasmepsin 2 (1LEE)	−6.7	2	Ser(A79)	2.70	H-Bond	Conventional
			Thr(A217)	2.99	H-Bond	Conventional
			Val(A78)	4.40	Hydrophobic	Pi-Alkyl/Alkyl
			Tyr(A77)	5.05	Hydrophobic	Pi-Alkyl/Alkyl
			Tyr(A77)	5.16	Hydrophobic	Pi-Alkyl/Alkyl
			Ile(A123)	4.66	Hydrophobic	Pi-Alkyl/Alkyl
			Ile(A32)	3.86	Hydrophobic	Pi-Alkyl/Alkyl
			Tyr(A192)	-	Electrostatic	Van der Waals
			Ser(A37)	-	Electrostatic	Van der Waals
			Asp(A34)		Electrostatic	Van der Waals
			Gly(A216)	-	Electrostatic	Van der Waals
			Ser(A218)	-	Electrostatic	Van der Waals
			Asp(A214)	-	Electrostatic	Van der Waals

## Data Availability

Data are presented in the manuscript.
